# Valor Prognóstico da Ultrassonografia Pulmonar para Resultados Clínicos em Pacientes com Insuficiência Cardíaca: Uma Revisão Sistemática e Metanálise

**DOI:** 10.36660/abc.20190662

**Published:** 2021-02-03

**Authors:** Yushu Wang, Di Shi, Fuqiang Liu, Ping Xu, Min Ma

**Affiliations:** 1 Chengdu City First People’s Hospital ChengduSichuan China Chengdu City First People’s Hospital , Chengdu , Sichuan - China; 2 Zigong Fourth People’s Hospital ZigongSichuan China Zigong Fourth People’s Hospital , Zigong , Sichuan - China; 3 Chengdu Sixth People’s Hospital Chengdu China Chengdu Sixth People’s Hospital , Chengdu - China

**Keywords:** Pulmão/Ultrassonografia, Linhas B, Prognóstico, Insuficiência Cardíaca, Revisão, Metanálise

## Abstract

**Fundamento:**

Existem informações conflitantes sobre se a ultrassonografia pulmonar avaliada por linhas B tem valor prognóstico em pacientes com insuficiência cardíaca (ICa).

**Objetivos:**

Avaliar o valor prognóstico da ultrassonografia pulmonar avaliada por linhas B em pacientes com ICa.

**Métodos:**

Quatro bases de dados (PubMed, EMBASE, Cochrane Library e Scopus) foram sistematicamente pesquisadas para identificar artigos relevantes. Reunimos a razão de risco (RR) e o intervalo de confiança de 95% (IC) de estudos elegíveis e realizamos análises de heterogeneidade, avaliação de qualidade e viés de publicação. Os dados foram agrupados usando um modelo de efeitos fixos ou de efeito aleatório. Um valor de p <0,05 foi considerado para indicar significância estatística.

**Resultados:**

Nove estudos envolvendo 1.212 participantes foram incluídos na revisão sistemática. As linhas B > 15 e > 30 na alta hospitalar foram significativamente associadas ao aumento do risco de desfecho combinado de mortalidade por todas as causas ou hospitalização por ICa (RR, 3,37, IC de 95%, 1,52-7,47; p = 0,003; RR, 4,01, IC de 95%, 2,29-7,01; p <0,001, respectivamente). O ponto de corte da linha B > 30 na alta foi significativamente associado ao aumento do risco de hospitalização por ICa (RR, 9,01, IC de 95%, 2,80-28,93; p <0,001). Além disso, o ponto de corte da linha B > 3 aumentou significativamente o risco de desfecho combinado de mortalidade por todas as causas ou hospitalização por ICa em pacientes ambulatoriais com ICa (RR, 3,21, IC de 95%, 2,09-4,93; I2 = 10%; p <0,00001).

**Conclusão:**

As linhas B podem predizer mortalidade por todas as causas e hospitalizações por ICa em pacientes com ICa. Outros grandes ensaios clínicos randomizados são necessários para explorar se lidar com as linhas B melhoraria o prognóstico nos ambientes clínicos. (Arq Bras Cardiol. 2020; [online].ahead print, PP.0-0)

## Introdução

A insuficiência cardíaca (ICa) continua sendo a principal causa de hospitalização nas últimas décadas devido a sua alta prevalência, morbidade e mortalidade. ^1^ A congestão pulmonar pode predizer tanto a mortalidade quanto a morbidade em pacientes com ICa, ^2^ e a descongestão é um dos principais objetivos da gerenciamento de ICa durante a hospitalização. ^3^

A ultrassonografia pulmonar (USP) é uma ferramenta simples, amigável para o paciente, confiável e sensível para detectar a congestão pulmonar avaliada por linhas B. ^4 , 5^ A linha B é um tipo de artefato de cauda de cometa que aparece como reverberação hiperecoica vertical discreta semelhante a lasers, surge da linha pleural, se estende até a parte inferior da tela, se move em sincronia com o deslizamento do pulmão e apaga as linhas A. ^6^ As linhas B representam septos interlobulares espessados. A soma das linhas B em todos os espaços escaneados produz uma pontuação que denota a extensão do fluido extravascular no pulmão, e zero é definido como uma ausência completa de linhas B na área investigada. ^7^ A USP à beira do leito foi reconhecida em uma declaração científica da *European Society of Cardiology* como um dos elementos-chave na mensuração da congestão clínica desde 2010, ^8^ e foi recomendada em 2015 para avaliar edema pulmonar em pacientes com suspeita de ICa aguda. ^9^

Uma técnica baseada em ultrassom para avaliar a congestão pulmonar tem servido como um auxílio na diferenciação das causas da dispneia aguda, principalmente em situações de acidente e emergência, ^10^ mas também como uma avaliação em outras condições. ^11 , 12^ Estudos em animais têm apoiado o uso da ultrassonografia torácica e detecção de linhas B como técnicas de diagnóstico de edema pulmonar cardiogênico em cães. ^13^ Além disso, ao USP foi identificada como uma ferramenta reprodutível e confiável para a detecção de congestão pulmonar, identificação do início de descompensação de ICa e avaliação da eficiência terapêutica para essa síndrome em camundongos. ^14^ As linhas B fornecem um biomarcador útil para avaliar o curso de tempo das alterações extravasculares da água do pulmão após as intervenções. Após o tratamento médico adequado da ICa, o padrão de linha B desaparece quase por completo, o que representa uma abordagem diagnóstica alternativa e fácil de usar para avaliar a congestão pulmonar em pacientes com ICa descompensada. ^15^ O número de linhas B mais alto foi associado a um risco aumentado de morbidade e mortalidade em outras configurações de doença, como a síndrome coronariana aguda ^16^ e a diálise. ^17^ No entanto, sua eficácia em pacientes com ICa não está bem estabelecida.

Devido ao número limitado de estudos clínicos sobre este tópico, acreditamos que vale a pena avaliar cuidadosamente as evidências acumuladas. Na presente meta-análise, examinamos sistematicamente o valor prognóstico da congestão pulmonar transmitida por linhas B em pacientes com ICa.

## Métodos

### Busca bibliográfica

Este estudo foi realizado sob a orientação da declaração de Itens de Relatório Preferidos para Revisões Sistemáticas e Metanálises ( *Preferred Reporting Items for Systematic Reviews and Meta-Analyses* – PRISMA). ^18^ A lista de verificação PRISMA 2009 foi listada no arquivo suplementar. Isso foi registrado com o PROSPERO (CRD 42019138780). Pesquisamos PubMed, EMBASE, *Cochrane Library* e *Scopus* desde sua data de início até julho de 2019 para identificar estudos elegíveis, usando palavras-chave e/ou termos de título de assunto médico como: “ *B lines* ” ou “ *lung ultrasound* ” ou “ *ultrasound lung comets* ” ou “ *pulmonary congestion* ”) e (“ *heart failure* ” ou “ *cardiac dysfunction* ” ou “ *cardiac failure* ” ou “ *cardiac insufficiency* ”. Não foram utilizadas restrições de idioma. As referências de literaturas relevantes também foram pesquisadas em busca de mais estudos elegíveis.

### Critérios de inclusão e exclusão do estudo

Os critérios de inclusão nesta revisão e metanálise fizeram referência aos participantes, intervenções, comparações, resultados e desenho do estudo (PICOS) conforme descrito no protocolo PRISMA, sendo:

1. inscrição de pacientes com ICa (seja de nova ICa ou agravamento da ICC exigindo hospitalização);
2. o uso de cometas pulmonares de ultrassom para avaliar a congestão pulmonar em pacientes com ICa;
3. razões de risco relatadas (RR) para medidas de resultados possíveis (mortalidade por todas as causas, hospitalização por ICa ou desfechos combinados); e
4. estudos de acompanhamento, incluindo análise post hoc de ensaios clínicos randomizados.

Os critérios de exclusão foram:

1. revisões, metanálises, estudo não-humano, cartas, relatos de caso e conferências; e
2. estudos que não fornecem resultados em pacientes com ICa.

### Extração de dados e avaliação de qualidade

Dois investigadores (Y.W. e X.P.) examinaram independentemente todos os títulos, resumos e artigos de texto completo extraídos de bancos de dados para estudos potencialmente relevantes. Qualquer discrepância foi resolvida por discussão entre todos os autores. Os seguintes dados foram extraídos de cada estudo: sobrenome do primeiro autor, ano de publicação, país onde o estudo foi realizado, os tipos de estudo envolvidos, o número de participantes, períodos de acompanhamento e desfechos de interesse. Uma escala de qualidade de Newcastle-Ottawa ( *Newcastle-Ottawa Quality scale* – NOS) variando de zero (mais baixa) a nove (mais alta) foi aplicada para avaliar a qualidade metodológica para estudos de coorte, conforme recomendado pelo *Cochrane Non-Randomized Studies Methods Working Group* . ^19^ Uma pontuação de ≥5 foi considerado de alta qualidade. Além disso, a ferramenta *Quality In Prognosis Studies* (QUIPS) foi aplicada para examinar o viés e a validade nos artigos de fatores prognósticos. ^20^

### Análise Estatística

Os *softwares* RevMan 5.3 ( *The Cochrane Collaboration* , Oxford) e Stata, versão 11 (StataCorp), foram usados adequadamente em todas as análises estatísticas. A estatística Cochrane Q e a estatística I ^2^ foram calculadas para avaliar a heterogeneidade entre os estudos. O teste estatístico Cochrane Q com um valor p ≤ 0,05 foi considerado estatisticamente significativo. Os valores de I ^2^ de 25, 50 e 75% corresponderam a graus de heterogeneidade baixo, moderado e alto, respectivamente. ^21^ Se I ^2^ fosse maior que 50%, optamos por usar um modelo de efeitos aleatórios (DerSimonian e método de Laird) para combinar os resultados e se I ^2^ fosse inferior a 50% criamos um modelo de efeitos fixos (método de Mantel-Haenszel). ^22^ O uso de um modelo de efeitos aleatórios também foi considerado quando o número de estudos era pequeno. Combinamos as razões de risco (RR) entre os estudos usando ponderação de variância inversa genérica e um intervalo de confiança de 95% (IC) para cada desfecho. O log geral (RR) com seu IC de 95% foi usado como o resumo do tamanho do efeito geral. Além disso, realizamos análises de subgrupos com base no número de linhas B na alta nos estudos incluídos. As análises de sensibilidade foram conduzidas excluindo um estudo envolvido nesta revisão e metanálise de cada vez para refletir o efeito do conjunto de dados específicos na RR geral. O viés de publicação foi analisado quantitativamente pelo teste de correlação de postos de Begg ^23^ e teste de regressão linear de Egger. ^24^ Um valor de p <0,05 foi considerado para indicar significância estatística.

## Resultados

### Resultados da Busca

**Figura 1:**
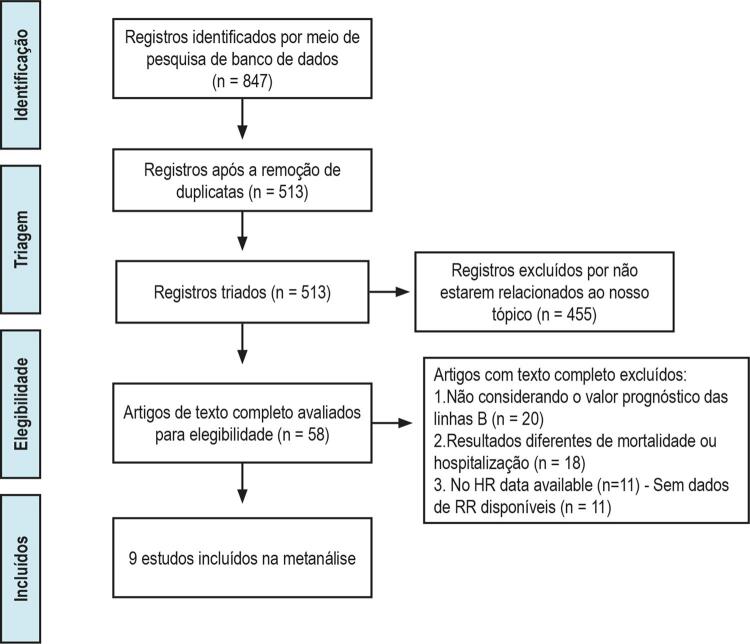
– Fluxograma do processo seletivo.

Nossa estratégia de busca foi delineada na Figura 1 . Nossa busca na literatura identificou 847 artigos potencialmente relevantes. Foram excluídos 455 estudos com base na triagem dos títulos e resumo desses artigos. Cinquenta e oito artigos foram excluídos após a revisão do texto completo e, finalmente, os 9 artigos restantes ^25 - 33^ foram incluídos na metanálise.

### Características do estudo e avaliação da qualidade

Os 9 estudos incluídos variaram de 54 a 342 pacientes, com uma população final de 1.212 pacientes. Destes, sete estudos foram realizados na Europa e um nos Estados Unidos. A Tabela 1 representa as características basais dos artigos incluídos nesta metanálise. Desses, oito estudos eram prospectivos ^25 - 30 , 32 , 33^ e um estudo retrospectivo. ^31^ Cinco de nove estudos ^27 , 29 , 30 , 32 , 33^ inscreveram um total de 792 pacientes ambulatoriais com ICa e os outros quatro estudos envolveram 420 pacientes hospitalizados por ICa. Além disso, quatro estudos ^26 , 28 , 31 , 32^ tiveram períodos de acompanhamento de 3 ou 4 meses e os outros cinco, de não menos de 6 meses. Os dados de hospitalização por ICa estavam disponíveis para apenas dois estudos, enquanto a maioria dos estudos relatou dados sobre o desfecho combinado de morte ou hospitalização por ICa. A idade média dos pacientes variou de 53 a 81 anos. Os pacientes nos estudos incluídos eram predominantemente do sexo masculino. As principais características dos pacientes foram resumidas na Tabela 2 . De acordo com a NOS apresentada na Tabela 3 , todos os estudos incluídos foram considerados de alta qualidade. No entanto, quatro artigos receberam pontuação 8 devido à duração relativamente curta do acompanhamento. A Tabela 4 mostra a avaliação da qualidade geral dos estudos incluídos usando a ferramenta QUIPS. Os sete artigos elegíveis eram geralmente de risco de viés baixo a moderado em termos de atrito de estudo, fator prognóstico e medição de resultados, participação no estudo, definição de resultados e análise estatística e relatórios. Além disso, alguns estudos apresentavam alto risco de viés porque relataram análise não ajustada ou não relataram análise ajustada.

**Tabela 1:** – Características principais dos estudos incluídos

Primeiro autor	Ano de publicação	País	Tipo de estudo	Participantes do estudo	Número de pacientes, n	Períodos de acompanhamento	Mortes por todas as causas, n	Hospitalização por ICa, n	Morte por todas as causas ou hospitalização por ICa, n	Desfechos reportados	Qualidade do estudo	Nível de significância adotado
Gargani ^25^	2015	Itália	Coorte prospectiva	Pacientes internados	100	6 meses	4	14	NA	Hospitalização por ICa	9	p <0,05
Coiro ^26^	2015	França	Coorte prospectiva	Pacientes internados	60	3 meses	10	15	18	Morte por todas as causas ou hospitalização por ICa	8	p <0,05
Gustafsson ^27^	2015	Suécia	Coorte prospectiva	Pacientes ambulatoriais	104	6 meses	14	18	24	Morte por todas as causas ou hospitalização por ICa	9	p <0,05
Cogliati ^28^	2016	Itália	Coorte prospectiva	Pacientes internados	150	100 dias	11	23	34	Morte por todas as causas ou hospitalização por ICa	8	p <0,05
Platz ^29^	2016	América	Coorte prospectiva	Pacientes ambulatoriais	195	6 meses	15	48	54	Morte por todas as causas ou hospitalização por ICa	9	p <0,05
Villanueva ^30^	2016	Espanha	Coorte prospectiva	Pacientes ambulatoriais	54	6 meses	NA	18	NA	Morte por todas as causas ou hospitalização por ICa	9	NR
Coiro ^31^	2016	França	Coorte retrospectiva	Pacientes internados	110	3 meses	16	26	33	Morte por todas as causas ou hospitalização por ICa	8	p <0,05
Miglioranza ^32^	2017	Brasil	Coorte prospectiva	Pacientes ambulatoriais	97	4 meses	3	23	NA	Morte por todas as causas, hospitalização por ICa, ECAM	8	p <0,05
Pellicori ^33^	2018	Reino Unido	Coorte prospectiva	Pacientes ambulatoriais	342	12 meses	25	35	NA	Morte por todas as causas ou hospitalização por ICa	9	p <0,05

*ICa: insuficiência cardíaca; ECAM: eventos cardíacos adversos maiores; NA: não aplicável; NR: não relatado.*

**Tabela 2:** – Características de base dos pacientes dos estudos incluídos

Estudos	Idade, média/mediana, anos	Homens, %	FEVE, média/mediana, %	Razão e/e'	DAC, %	HTN, %	DM, %	IECA/BRA, %	β-bloqueadores, %	ARM, %	Diuréticos, %	Digoxina, %
Gargani 2015	70	73	37	NA	NA	57	39	63	60	60	100	NA
Coiro 2015	72	68	38	19,11 ± 9,5	32	NA	NA	NA	NA	NA	NA	NA
Gustafsson 2015	72	72	NA	NA	40	57	24	95	89	31	78	NA
Cogliati 2016	81	42	48	NA	42	62	34	69	66	39	96	24
Platz 2016	NA	61	32	NA	NA	71	49	67	89	29	92	21
María 2016	79	54	NA	NA	33	94	54	72	57	NA	100	17
Coiro 2016	73	55	39	16 ± 1	46	NA	NA	NA	NA	NA	NA	NA
Miglioranza 2017	53	61	28	17 (13,30)	30	53	23	66	95	53	62	50
Pellicori 2018	NA	67	NA	NA	49	55	29	85	73	49	75	NA

*FEVE: fração de ejeção do ventrículo esquerdo; DAC: doença arterial coronariana; HTN: hipertensão; DM: diabetes mellitus; IECA: inibidor da enzima de conversão da angiotensina; BRA: bloqueador do receptor da angiotensina; ARM: antagonista do receptor mineralocorticóide; NA: não aplicável.*

**Tabela 3:** – Avaliação da qualidade do estudo usando a Escala de Newcastle-Ottawa para estudos de coorte

	Seleção					Desfecho			
Primeiro autor, ano de publicação (referência)	Representatividade da coorte exposta	Seleção da coorte não exposta	Apuração da exposição	Resultado de interesse ausente no início do estudo	Comparabilidade	Avaliação de desfechos	Acompanhamento por tempo suficiente para que os resultados ocorram	Adequação do acompanhamento	Pontuação total
Gargani 2015	*	*	*	*	* *	*	*	*	9
Coiro 2015	*	*	*	*	* *	*	-	*	8
Gustafsson 2015	*	*	*	*	* *	*	*	*	9
Cogliati 2016	*	*	*	*	* *	*	-	*	8
Platz 2016	*	*	*	*	* *	*	*	*	9
Villanueva 2016	*	*	*	*	* *	*	*	*	9
Coiro 2016	*	*	*	*	* *	*	-	*	8
Miglioranza 2017	*	*	*	*	* *	*	-	*	8
Pellicori 2018 ^33^	*	*	*	*	* *	*	*	*	9

Asteriscos são as classificações por estrelas de acordo com a Escala de Newcastle-Ottawa; * e ** indicam as classificações mais altas para essas categorias.

**Tabela 4:** – Avaliação da qualidade do nível do estudo usando a ferramenta Quality In Prognosis Studies

Estudo	Participação no estudo	Atrito de estudo	Medição do fator prognóstico	Medição de resultado	Viés de confusão do estudo	Análise estatística e relatórios
Gargani 2015 ^25^	B	B	B	B	A	B
Coiro 2015 ^26^	B	M	B	B	A	B
Gustafsson 2015 ^27^	B	B	B	B	B	B
Cogliati 2016 ^28^	B	B	B	B	A	B
Platz 2016 ^29^	B	B	B	B	B	B
Villanueva 2016 ^30^	B	B	M	B	A	B
Coiro 2016 ^31^	B	M	B	B	B	B
Miglioranza 2017 ^32^	B	B	B	B	B	B
Pellicori 2018 ^33^	B	B	B	B	B	B

*B: baixo; M: moderado; A: alto*

### Linhas B de alta hospitalar e desfecho combinado de mortalidade por todas as causas ou hospitalização por ICa

**Figura 2:**
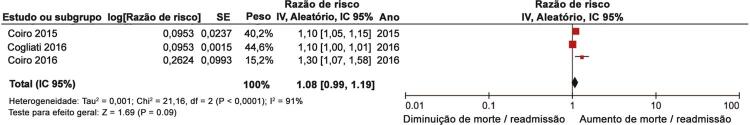
– Gráficos para linhas B de alta hospitalar e desfecho combinado de mortalidade por todas as causas ou hospitalização por ICa.

**Figura 3:**
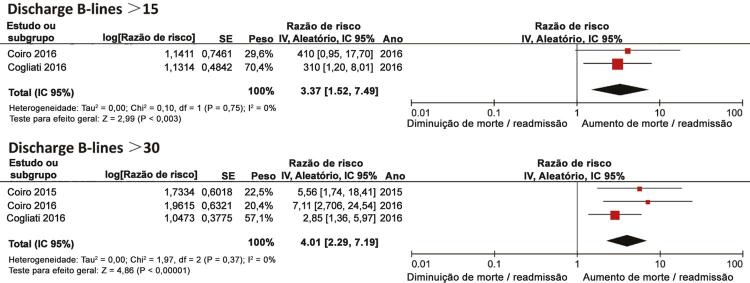
Análise de subgrupo das linhas B de alta e desfecho combinado de mortalidade por todas as causas ou hospitalização por ICa.

Três estudos ^26 , 28 , 31^ relataram a associação entre linhas B de alta hospitalar e desfecho combinado de óbito ou hospitalização por ICa. Estimativas agrupadas mostraram que houve uma forte tendência para a associação entre linhas B de alta e aumento do risco de desfecho combinado de morte ou hospitalização por ICa (RR, 1,08, IC de 95%, 0,99-1,19; I ^2^ = 91%; P = 0,09 ; Figura 2 ). A análise de subgrupo ^28 , 31^ com base no número de linhas B na alta revelou que as linhas B > 15 na alta foram significativamente associadas ao aumento do risco de morte ou hospitalização por ICa (RR, 3,37, IC 95%, 1,52-7,47; I ^2^ = 0%; p = 0,003; Figura 3 ). Além disso, as linhas B > 30 na alta se correlacionaram significativamente com aumento do risco de desfecho combinado de morte ou hospitalização por ICa (RR, 4,01, IC 95%, 2,29-7,01; I2 = 0%; p <0,001; Figura 3 ). A análise de sensibilidade restrita a dois estudos prospectivos ^26 , 28^ demonstrou que as linhas B > 30 se correlacionaram significativamente com o desfecho combinado de morte ou hospitalização por ICa (RR, 3,46, IC 95%, 1,86-6,47; I ^2^ = 0%; p = 0,0001). A análise de sensibilidade pela omissão de qualquer estudo único produziu resultados semelhantes.

### Linhas B de alta hospitalar e hospitalização por ICa

**Figura 4:**
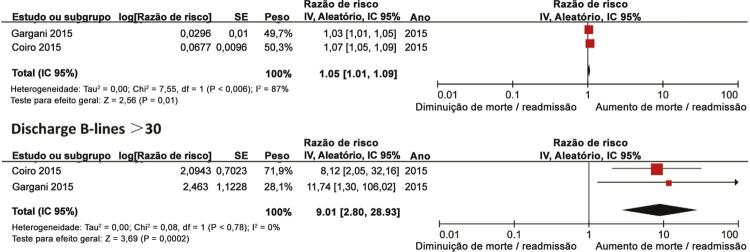
– Gráficos para linhas B e hospitalização por ICa.

Dois estudos ^25 , 26^ relataram a associação entre linhas B de alta e hospitalização por ICa. As estimativas gerais demonstraram que as linhas B de alta foram significativamente associadas à hospitalização por ICa (RR, 1,05, IC 95%, 1,01-1,09; p = 0,01; Figura 4 ), com heterogeneidade substancial (I ^2^ = 87%). Além disso, a análise de subgrupo indicou que as linhas B > 30 na alta aumentaram significativamente o risco de hospitalização por ICa (RR, 9,01, IC 95%, 2,80-28,93; p <0,001; Figura 4 ), sem heterogeneidade (I ^2^ = 0%).

### Linhas B e desfechos combinados de morte e hospitalização por ICa em pacientes ambulatoriais com ICa

**Figura 5:**
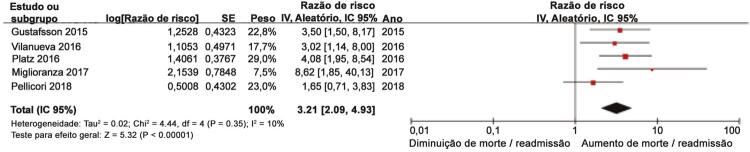
– Gráficos para linhas B e desfecho combinado de mortalidade por todas as causas ou hospitalização por ICa em pacientes ambulatoriais com ICa.

Cinco estudos ^27 , 29 , 30 , 32 , 33^ avaliaram a associação entre linhas B e desfecho combinado de morte e hospitalização por ICa em pacientes ambulatoriais com ICa. As RR agrupadas mostraram que as linhas B > 3 aumentaram significativamente o risco de desfecho combinado de morte ou hospitalização por ICa em pacientes ambulatoriais com ICa (RR, 3,21, IC 95%, 2,09-4,93; I ^2^ = 10%; p <0,00001; Figura 5 ). A análise de sensibilidade restrita a três estudos ^27 , 30 , 32 , 33^ conduzidos fora da América demonstrou que as linhas B > 3 se correlacionaram significativamente com o desfecho combinado de morte ou hospitalização por ICa (RR, 2,96, IC 95%, 1,69-5,16; I ^2^ = 22%; p <0,001). A análise de sensibilidade foi conduzida pela omissão de qualquer estudo único que não alterou significativamente as estimativas de efeito geral.

### Viés de publicação

Os testes de Egger e Begg não sugeriram viés de publicação significativo de desfecho combinado de morte ou hospitalização por ICa em pacientes internados (Egger p = 0,15 e Begg p = 1,00) e ambulatoriais (Egger p = 0,33 e Begg p = 1,0).

## Discussão

A presente metanálise indicou que em pacientes com ICa, os pontos de corte das linhas B > 15 e > 30 na alta foram preditivas do desfecho composto de mortalidade por todas as causas ou readmissão por ICa em pacientes hospitalizados. Além disso, um ponto de corte de linha B > 30 na alta foi preditivo de hospitalização por ICa. Em pacientes ambulatoriais com ICa, as linhas B > 3 previram fortemente o desfecho composto de mortalidade por todas as causas ou readmissão por ICa. Dada a heterogeneidade entre os estudos incluídos e o tamanho limitado da amostra, esses resultados devem ser considerados como geradores de hipóteses para pesquisas futuras.

Uma revisão sistemática recente sugeriu que muitas linhas B em pacientes com ICa descompensada identificaram alto nível de risco para eventos adversos. ^34^ No entanto, essa revisão consistiu em apenas cinco estudos sobre avaliação do valor prognóstico da USP na ICa e não realizou metanálise com base em diferentes números de linhas B na alta. Outra revisão apoiou o uso de USP no tratamento da ICa descompensada aguda, tanto como modalidade diagnóstica quanto no monitoramento da terapia para ICa. ^35^ Em um ambulatório de ICa sistólica moderada a grave, um estudo demonstrou que as linhas B estavam significativamente associadas a mais parâmetros clinicamente estabelecidos de descompensação, como a porção N-terminal do peptídeo natriurético tipo B (NT-proBNP), a pontuação de congestão clínica e razão E/e’, e a pontuação de corte da linha B ≥ 15 sugeriram descompensação de ICa. ^36^ No entanto, o valor prognóstico de linhas B, incremental aos fatores de risco bem como àqueles indicadores estabelecidos de congestão clínica em pacientes com ICa, requer investigação adicional.

Existem poucos dados que descrevem as características das linhas B e suas diferenças em pacientes com ICa com função sistólica ventricular preservada (HFpEF) e reduzida (HFrEF). Os estudos incluídos envolveram pacientes com ICa, mas demonstraram seus resultados sem estratificação por FE. Embora a congestão melhore substancialmente durante a hospitalização em resposta à terapia padrão como único método, pacientes com HFrEF e sinais e sintomas de repouso ausentes ou mínimos avaliados pelo BNP e a pontuação de congestão clínica ainda experimentaram uma alta taxa de mortalidade e readmissão. ^37^ É importante notar que o estudo por Coiro et al. demonstrou que a adição de ≥ 15 e ≥ 30 linhas B ao BNP e ao padrão *New York Health Association* (NYHA) melhoraram a classificação de risco e as linhas B previram de maneira independente a mortalidade e hospitalização por ICa. ^26^ A ausência ou uma pequena quantidade de linhas B identificou aqueles com risco extremamente baixo de ICa por re-hospitalização, mas se lidar ou não com essa congestão pulmonar residual melhoraria o desfecho do paciente deve ser objeto de investigação adicional. ^38^

O padrão ouro ainda não foi estabelecido para a avaliação quantitativa da congestão pulmonar. É importante observar que o posicionamento do paciente pode afetar o número de linhas B em pacientes com ICa, por exemplo, o número de linhas B foi menor na posição sentada do que na posição supinada. ^39^ Além disso, dois estudos ^25 , 27^ incluídos nesta revisão e metanálise usaram ambos os métodos das 28 e 8 regiões de varredura para exames de USP. Esses dois métodos foram recomendados como úteis na avaliação do edema pulmonar. ^40^ No entanto, ao relatar os achados de USP, é importante que os dados contínuos e categóricos sejam padronizados para apresentar medidas de USP (por exemplo, número de regiões pulmonares) para facilitar a comparação de resultados em estudos de ICa. No presente trabalho, os estudos incluídos indicaram o valor prognóstico das linhas B em pacientes com ICa internados e ambulatoriais. No entanto, devido aos diferentes desfechos de interesse (hospitalização por ICa *versus* desfechos combinados de hospitalização e mortalidade) e diferentes períodos de acompanhamento clínico (3 *versus* 6 meses), há uma ligeira diferença no ponto de corte ideal relatado para as linhas B; entretanto, eles variaram entre 15 e 30. Ensaios clínicos randomizados maiores são necessários para investigar até que ponto o uso de USP beneficiaria pacientes com ICa. Além disso, mais estudos são necessários para descobrir se a USP pode ser usada para identificar diferentes fenótipos de pacientes com ICa e para ser adaptada às necessidades individuais do paciente.

### Limitações

Devido ao seu desenho, nossa análise não permitiu a demonstração da superioridade das linhas B em comparação com outros biomarcadores de ICa, como a classificação NYHA, o NT-proBNP ou o teste de caminhada de 6 min, nem avaliamos o valor prognóstico incremental das linhas B sobre os marcadores estabelecidos para congestão. Além disso, até onde sabemos, embora estejamos fornecendo a primeira revisão e metanálise das linhas B em pacientes com ICa, mais estudos são necessários para o tratamento ideal de pacientes com ICa no que diz respeito ao valor integrativo das linhas B associado ao BNP ou a fatores de risco. Em terceiro lugar, existe uma heterogeneidade substancial nesta revisão e metanálise entre os estudos. Os artigos incluídos com diferentes características dos pacientes, quantificação de linhas B e risco de viés podem contribuir para a heterogeneidade entre os estudos. Além disso, o número de pacientes incluídos em nossa metanálise foi relativamente pequeno, o que pode ter impacto na quantificação exata do valor prognóstico das linhas B. Além disso, os estudos incluídos consideraram diferentes desfechos. Apenas um estudo ^24^ forneceu valores de linhas B tanto na admissão quanto na alta para o desfecho combinado de mortalidade por todas as causas ou hospitalização por ICa. Seria interessante examinar as mudanças entre os números ou posições das linhas B na admissão e antes da alta.

## Conclusões

A presente metanálise demonstrou que as linhas B podem predizer mortalidade por todas as causas e hospitalizações por ICa em pacientes com ICa. Outros grandes ensaios clínicos randomizados são necessários para explorar se lidar com as linhas B melhoraria o prognóstico nos ambientes clínicos.
