# Supplementary material for: Valor Prognóstico da Ultrassonografia Pulmonar para Resultados Clínicos em Pacientes com Insuficiência Cardíaca: Uma Revisão Sistemática e Metanálise
Source: Arq Bras Cardiol. 2021 Feb 3;116(3):383–92. [Article in Portuguese] doi: 10.36660/abc.20190662 (PMC8159549; doi:10.36660/abc.20190662)
Supplement: Translation

# Translation: Prognostic Value of Lung Ultrasound for Clinical Outcomes in Heart Failure Patients: A Systematic Review and Meta-Analysis

**Authors:** Yushu Wang, Di Shi, Fuqiang Liu, Ping Xu, Min Ma

**Affiliations:** 1 China Chengdu City First People’s Hospital, Chengdu, Sichuan - China; 2 China Zigong Fourth People’s Hospital, Zigong, Sichuan - China; 3 China Chengdu Sixth People’s Hospital, Chengdu - China

**Keywords:** Lung/ultrassonography, B Lines, Prognosis, Heart Failure, Review, Meta-Analysis

## Abstract

**Background:**

There is conflicting information about whether lung ultrasound assessed by B-lines has prognostic value in patients with heart failure (HF).

**Objectives:**

To evaluate the prognostic value of lung ultrasound assessed by B-lines in HF patients.

**Methods:**

Four databases (PubMed, EMBASE, Cochrane Library, and Scopus) were systematically searched to identify relevant articles. We pooled the hazard ratio (HR) and 95% confidence interval (CI) from eligible studies and carried out heterogeneity, quality assessment, and publication bias analyses. Data were pooled using a fixed-effects or random-effect model. A p value < 0.05 was considered to indicate statistical significance.

**Results:**

Nine studies involving 1,212 participants were included in the systematic review. B-lines > 15 and > 30 at discharge were significantly associated with increased risk of combined outcomes of all-cause mortality or HF hospitalization (HR, 3.37, 95% CI, 1.52-7.47; p = 0.003; HR, 4.01, 95% CI, 2.29-7.01; p < 0.001, respectively). A B-line > 30 cutoff at discharge was significantly associated with increased risk of HF hospitalization (HR, 9.01, 95% CI, 2.80-28.93; p < 0.001). Moreover, a B-line > 3 cutoff significantly increased the risk for combined outcomes of all-cause mortality or HF hospitalization in HF outpatients (HR, 3.21, 95% CI, 2.09-4.93; I2 = 10%; p < 0.00001).

**Conclusion:**

B-lines could predict all-cause mortality and HF hospitalizations in patients with HF. Further large randomized controlled trials are needed to explore whether dealing with B-lines would improve the prognosis in clinical settings.

## Introduction

Heart failure (HF) remains the leading cause of hospitalization in recent decades due to its high prevalence, morbidity, and mortality rates.^1^Pulmonary congestion can predict both mortality and morbidity in patients with HF,^2^and decongestion is one of the primary goals of HF management in patients during hospitalization.^3^

Lung ultrasound (LUS) is a simple, patient-friendly, reliable, sensitive tool to detect pulmonary congestion assessed by B-lines.^4 , 5^B-line is a kind of comet-tail artifact that appears as discrete laser-like vertical hyperechoic reverberation artifacts, arises from the pleural line, extends to the bottom of the screen, moves synchronously with lung sliding and erases A-lines.^6^B-lines represent thickened interlobular septa. The sum of B-lines in all scanned spaces yields a score denoting the extent of extravascular fluid in the lung, and zero is defined as a complete absence of B-lines in the investigated area.^7^Bedside LUS has been recognized in a scientific statement of the European Society of Cardiology as one of the key elements in the measurement of clinical congestion since 2010,^8^and was recommended in 2015 to assess pulmonary edema in patients with suspected acute HF.^9^

An ultrasound-based technique to evaluate pulmonary congestion has served as an aid in the differentiating causes of acute dyspnea mainly in accident and emergency setting,^10^but also as an evaluation in other conditions.^11 , 12^Animal studies have supported the use of thoracic ultrasonography and detection of B-lines as techniques for diagnosing cardiogenic pulmonary edema in dogs.^13^Also, LUS has been identified to be a reproducible as well as a reliable tool to detect pulmonary congestion, to identify the onset of HF decompensation, and to evaluate the therapeutic efficiency for this syndrome in mice.^14^B-lines provide a useful biomarker to evaluate the time course of extra-vascular lung water changes after interventions. After adequate HF medical treatment, B-line pattern mostly clears, which represents an easy-to-use alternative bedside diagnostic approach to evaluate pulmonary congestion in patients with decompensated HF.^15^A higher B-line number was associated with an increased risk of morbidity and mortality in other disease settings such as acute coronary syndrome^16^and dialysis.^17^However, its efficacy in patients with HF has not been well established.

Owing to the limited number of clinical studies on this topic, we believed it worthwhile to carefully evaluate the accumulated evidence. In the present meta-analysis, we systematically examined the prognostic value of pulmonary congestion conveyed by B-lines in patients with HF.

## Methods

### Literature search

This study was performed under the guidance of the Preferred Reporting Items for Systematic Reviews and Meta-Analyses (PRISMA) statement.^18^The PRISMA 2009 checklist was listed in the supplementary file. This was registered with PROSPERO (CRD 42019138780). We searched PubMed, EMBASE, Cochrane Library and Scopus from their start date up to July 2019 to identify eligible studies, using the keywords and/or medical subject heading terms: “B lines” or “lung ultrasound” or “ultrasound lung comets ” or “pulmonary congestion”) and (“heart failure” or “cardiac dysfunction” or “cardiac failure” or “cardiac insufficiency”. No language restrictions were used. The references of relevant literatures were also searched to find more eligible studies.

### Study inclusion and exclusion criteria

The inclusion criteria in this review and meta-analysis were as follows with reference to participants, interventions, comparisons, outcomes, and study design (PICOS) as described on PRISMA protocol:

1. enrollment of patients with HF (either of new HF or worsening chronic heart failure requiring hospitalization);
2. use of ultrasound lung comets to assess pulmonary congestion in HF patients;
3. reported hazard ratios (HR) for possible outcome measures (all-cause mortality, hospitalization by HF, or combined outcomes); and
4. follow-up studies, including post hoc analysis of randomized clinical trials.

The exclusion criteria were:

1. reviews, meta-analyses, non-human study, letters, case reports, and conferences; and
2. studies that do not provide results on patients with HF.

### Data extraction and quality assessment

Two investigators (Y.W. and X.P.) independently examined all titles, abstracts and full-text articles extracted from databases for potentially relevant studies. Any discrepancies were resolved by discussion among all authors. Data extracted from each study were: first author’s last name, year of publication, country where the study was carried out, the types of study involved, the number of participants, follow-up periods, and outcomes of interest. A Newcastle-Ottawa Quality scale (NOS) ranging from zero (lowest) to nine (highest) was applied to assess the methodological quality for cohort studies, as recommended by the Cochrane Non-Randomized Studies Methods Working Group.^19^A score of ≥5 was considered to be of high quality. In addition, the Quality In Prognosis Studies (QUIPS) tool was applied to examine bias and validity in articles of prognostic factors.^20^

### Statistical analysis

The RevMan 5.3 (The Cochrane Collaboration, Oxford) and Stata version 11 (StataCorp) software were properly used in all statistical analyses. The Cochrane Q and the I^2^statistics were calculated to assess heterogeneity across the studies. The Cochrane Q-statistic test with a p-value ≤ 0.05 was considered statistically significant. I^2^values of 25, 50, and 75% corresponded to low, moderate, and high degrees of heterogeneity, respectively.^21^If I^2^was greater than 50%, we chose to use a random-effects model (DerSimonian and Laird’s method) to combine the results and if I^2^was lower than 50% we created a fixed-effects model (Mantel-Haenszel’s method).^22^The use of a random-effects model was also considered when the number of studies was small. We combined the HR across studies using generic inverse-variance weighting and the 95% confidence interval (CI) for each outcome. The overall log (HR) with its 95%CI was used as the summary of the overall effect size. In addition, subgroup analyses were carried out based on numbers of B-lines at discharge in the included studies. Sensitivity analyses were conducted by excluding one study involved in this review and meta-analysis at a time to reflect the effect of the specific data set on the overall HR. Publication bias was quantitatively analyzed by the Begg’s rank correlation test^23^and the Egger’s linear regression test.^24^A p-value < 0.05 was considered to indicate statistical significance.

## Results

### Search Results

Our search strategy was outlined in Figure 1 . Our literature search identified 847 potentially relevant articles. We excluded 455 studies based on the screening of titles and abstracts of those papers. Fifty-eight articles were excluded after going through full-text review, and finally the remaining 9 articles^25 - 33^were included in the meta-analysis.

**Figure 1:** – Flow diagram of selection process.

### Study characteristics and quality assessment

The 9 studies included here ranged from 54 to 342 patients, with a final population of 1,212 patients. Of these, seven studies were carried out in Europe and one in the United States. Table 1 represents the baseline characteristics of the articles included in this meta-analysis. Of those, there were eight prospective studies^25 - 30 , 32 , 33^and one retrospective one.^31^Five out of nine studies^27 , 29 , 30 , 32 , 33^enrolled a total of 792 HF outpatients and the other four studies enrolled 420 patients hospitalized for HF. In addition, four studies^26 , 28 , 31 , 32^had follow-up durations of 3 or 4 months and the other five studies had follow-up periods of no less than 6 months. Data for HF hospitalization was available for only two studies, while most studies reported data on combined outcomes of death or HF hospitalization. The mean age of patients ranged from 53 to 81 years old. The patients in the included studies were predominately male. The main patients’ characteristics were summarized in Table 2 . According to the NOS shown in Table 3 , all of th included studies were considered to be of high-quality. However, four articles were given a score of 8 due to relatively short follow-up duration. Table 4 showed the overall quality assessment of the included studies using the QUIPS tool. The seven eligible articles were usually at low to moderate risk of bias in terms of study attrition, prognostic factor and outcome measurement, study participation, definition of outcomes and statistical analysis and reporting. Furthermore, some studies were at high risk of bias because they reported unadjusted analysis or did not report adjusted analysis.

**Table 1:** – Key characteristics of the included studies

First Author	Publication year	Country	Type of study	Study participants	Number of patients, n	Follow-up periods	All-cause deaths, n	HF hospitalization, n	All-Cause death or HF hospitalization, n	Reported outcomes	Quality of study	Level of significance adopted
Gargani^25^	2015	Italy	Prospective cohort	Inpatients	100	6 months	4	14	NA	HF hospitalization	9	p <0.05
Coiro^26^	2015	France	Prospective cohort	Inpatients	60	3 months	10	15	18	All-cause death or HF hospitalization	8	p <0.05
Gustafsson^27^	2015	Sweden	Prospective cohort	Outpatients	104	6 months	14	18	24	All-cause death or HF hospitalization	9	p <0.05
Cogliati^28^	2016	Italy	Prospective cohort	Inpatients	150	100 days	11	23	34	All-cause death or HF hospitalization	8	p <0.05
Platz^29^	2016	America (USA?)	Prospective cohort	Outpatients	195	6 months	15	48	54	All-cause death or HF hospitalization	9	p <0.05
Villanueva^30^	2016	Spain	Prospective cohort	Outpatients	54	6 months	NA	18	NA	All-cause death or HF hospitalization	9	NR
Coiro^31^	2016	France	Retrospective cohort	Inpatients	110	3 months	16	26	33	All-cause death or HF hospitalization	8	p <0.05
Miglioranza^32^	2017	Brazil	Prospective cohort	Outpatients	97	4 months	3	23	NA	All-cause death, HF hospitalization, MACE	8	p <0.05
Pellicori^33^	2018	United Kingdom	Prospective cohort	Outpatients	342	12 months	25	35	NA	All-cause death or HF hospitalization	9	p <0.05

*HF: heart failure; MACE: major adverse cardiac events; NA: not applicable; NR: not reported.*

**Table 2:** – Baseline characteristics of patients from the included studies

Studies	Age, mean/median, years	Men, %	LVEF, mean/median, %	E/e' ratio	CAD, %	HTN, %	DM, %	ACE-I/ARB, %	β-blockers, %	MRA, %	Diuretics, %	Digoxin, %
Gargani 2015	70	73	37	NA	NA	57	39	63	60	60	100	NA
Coiro 2015	72	68	38	19.11 ± 9.5	32	NA	NA	NA	NA	NA	NA	NA
Gustafsson 2015	72	72	NA	NA	40	57	24	95	89	31	78	NA
Cogliati 2016	81	42	48	NA	42	62	34	69	66	39	96	24
Platz 2016	NA	61	32	NA	NA	71	49	67	89	29	92	21
María 2016	79	54	NA	NA	33	94	54	72	57	NA	100	17
Coiro 2016	73	55	39	16 ± 1	46	NA	NA	NA	NA	NA	NA	NA
Miglioranza 2017	53	61	28	17 (13.30)	30	53	23	66	95	53	62	50
Pellicori 2018	NA	67	NA	NA	49	55	29	85	73	49	75	NA

*LVEF: left ventricular ejection fraction; CAD: coronary artery disease; HTN: hypertension; DM: diabetes mellitus; ACEI: angiotensin-converting enzyme inhibitor; ARB: angiotensin receptor blocker; MRA: mineralocorticoid receptor antagonist; NA: not applicable.*

**Table 3:** – Study quality assessment using the Newcastle-Ottawa Scale for cohort studies

	Selection					Outcome			
First author, year of publication (reference)	Representativeness of exposed cohort	Selection of nonexposed cohort	Ascertainment of exposure	Outcome of interest absent at start of study	Comparability	Assessment of outcome	Follow-up long enough for outcomes to occur	Adequacy of follow-up	Total score
Gargani 2015	*	*	*	*	* *	*	*	*	9
Coiro 2015	*	*	*	*	* *	*	-	*	8
Gustafsson 2015	*	*	*	*	* *	*	*	*	9
Cogliati 2016	*	*	*	*	* *	*	-	*	8
Platz 2016	*	*	*	*	* *	*	*	*	9
Villanueva 2016	*	*	*	*	* *	*	*	*	9
Coiro 2016	*	*	*	*	* *	*	-	*	8
Miglioranza 2017	*	*	*	*	* *	*	-	*	8
Pellicori 2018^33^	*	*	*	*	* *	*	*	*	9

Asterisks are the star ratings per Newcastle-Ottawa Scale; * and ** indicate the highest ratings for these categories.

**Table 4:** – Study-level quality assessment using the Quality in Prognosis Studies tool

Study	Study participation	Study attrition	Prognostic factor measurement	Outcome measurement	Study confounding	Statistical analysis and reporting
Gargani 2015^25^	L	L	L	L	H	L
Coiro 2015^26^	L	M	L	L	H	L
Gustafsson 2015^27^	L	L	L	L	L	L
Cogliati 2016^28^	L	L	L	L	H	L
Platz 2016^29^	L	L	L	L	L	L
Villanueva 2016^30^	L	L	M	L	H	L
Coiro 2016^31^	L	M	L	L	L	L
Miglioranza 2017^32^	L	L	L	L	L	L
Pellicori 2018^33^	L	L	L	L	L	L

*.L: low; M: moderate; H: high*

### Discharge B-lines and combined outcomes of all-cause mortality or HF hospitalization

Three studies^26 , 28 , 31^reported the association between discharge B-lines and combined outcomes of death or HF hospitalization. Pooled estimates showed that there was a strong tendency toward the association between discharge B-lines and increased risk of combined outcomes of death or HF hospitalization (HR, 1.08, 95% CI, 0.99-1.19; I^2^= 91%; p = 0.09; Figure 2 ). Subgroup analysis^28 , 31^based on numbers of B-lines at discharge revealed that B-lines > 15 at discharge was significantly associated with increased risk of death or HF hospitalization (HR, 3.37, 95% CI, 1.52-7.47; I^2^= 0%; p = 0.003; Figure 3 ). Also, B-lines > 30 at discharge significantly correlated with increased risk of combined outcomes of death or HF hospitalization (HR, 4.01, 95% CI, 2.29-7.01; I^2^= 0%; p < 0.001; Figure 3 ). Furthermore, sensitivity analysis restricted to two prospective studies^26 , 28^demonstrated that B-lines > 30 significantly correlated with combined outcomes of death or HF hospitalization (HR, 3.46, 95% CI, 1.86-6.47; I^2^= 0%; p = 0.0001). Sensitivity analysis by omitting any single study yielded similar results.

**Figure 2:** – Forest plots for discharge B-lines and combined outcomes of all-cause mortality or HF hospitalization.

**Figure 3:** – Subgroup analysis of discharge B-lines and combined outcomes of all-cause mortality or HF hospitalization.

### Discharge B-lines and HF hospitalization

Two studies^25 , 26^reported the association between discharge B-lines and HF hospitalization. Overall estimates demonstrated that discharge B-lines were significantly associated with HF hospitalization (HR, 1.05, 95% CI, 1.01-1.09; p = 0.01; Figure 4 ), with substantial heterogeneity (I^2^= 87%). Furthermore, subgroup analysis indicated that B-lines > 30 at discharge significantly increased risk of HF hospitalization (HR, 9.01, 95% CI, 2.80-28.93; p < 0.001; Figure 4 ), with no heterogeneity (I^2^= 0%).

**Figure 4:** – Forest plots for B-lines and HF hospitalization

### B-lines and combined outcomes of all-cause mortality or HF hospitalization in HF outpatients

Five studies^27 , 29 , 30 , 32 , 33^assessed the association between B-lines and combined outcomes of death and HF hospitalization in HF outpatients. The pooled HRs showed that B-lines > 3 significantly increased the risk for combined outcomes of death or HF hospitalization in HF outpatients (HR, 3.21, 95% CI, 2.09-4.93; I^2^= 10%; p < 0.00001; Figure 5 ). Sensitivity analysis restricted to three studies^27 , 30 , 32 , 33^conducted outside of America demonstrated that B-lines > 3 significantly correlated with combined outcomes of death or HF hospitalization (HR, 2.96, 95% CI, 1.69-5.16; I^2^= 22%; p < 0.001). Sensitivity analysis was further conducted by omitting any single study that did not significantly alter the overall effect estimates.

**Figure 5:** – Forest plots for B-lines and combined outcomes of all-cause mortality or HF hospitalization in HF outpatients.

### Publication bias

Egger’s and Begg’s tests suggested no significant publication bias of combined outcomes of death or HF hospitalization in both in- (Egger p = 0.15 and Begg p = 1.00) and outpatients (Egger p = 0.33 and Begg p = 1.0).

## Discussion

The present meta-analysis indicated that, in patients with HF, B-lines >15 and >30 cutoff at discharge were predictive of the composite outcome of all-cause mortality or HF readmission in hospitalized patients. Additionally, a B-line >30 cutoff at discharge was predictive of HF hospitalization. In HF outpatients, B-lines >3 strongly predicted the composite outcomes of all-cause mortality or HF readmission. Given the heterogeneity across the included studies and limited sample size, these findings should be considered as hypothesis-generating for future research.

A recent systematic review suggested that plenty of B-lines in patients with decompensated HF identified that those were at high level of risk for adverse events.^34^However, this review consisted of only five studies evaluating the prognostic value of LUS in HF and did not perform meta-analysis based on different numbers of B-lines at discharge. Another review supported the use of LUS in the management of acute decompensated HF, both as a diagnostic modality and in monitoring HF therapy.^35^In a moderate to severe systolic HF outpatient clinic, a study demonstrated that B-lines were significantly associated with more clinically established parameters of decompensation, such as the amino-terminal portion of B-type natriuretic peptide (NT-proBNP), clinical congestion score and E/e’ ratio, and B-line ≥15 cutoff suggested HF decompensation.^36^However, the prognostic value of B-lines that is incremental to risk factors as well as those established indicators of clinical congestion in HF patients require further investigation.

There is a paucity of data describing features of B-lines and their differences in HF patients with preserved (HFpEF) and reduced (HFrEF) ventricular systolic function. The included studies enrolled HF patients but demonstrated their results without stratification by EF. Although congestion improves substantially during hospitalization in response to standard therapy alone, patients with HFrEF and with absent or minimal resting signs and symptoms at discharge evaluated by BNP and clinical congestion score still experienced high mortality and readmission rates.^37^Importantly, the study by Coiro et al. demonstrated that the addition of ≥15 and ≥30 B-lines to BNP and the New York Heart Association (NYHA) class had improved risk classification, and B-lines independently predicted mortality and hospitalization for HF.^26^The absence or a small amount of B-lines identified those at extremely low risk of HF rehospitalization, but whether dealing with this residual pulmonary congestion would improve patient outcome should be the issue of further investigation.^38^

The gold standard has not yet been established for the quantitative assessment of pulmonary congestion. Of note, patient positioning may affect the number of B-lines in HF patients, for example, the number of B-lines was lower in the sitting than in the supine position.^39^Moreover, two studies^25 , 27^included in this review and meta-analysis used both methods of the 28 and 8 scanning regions for LUS examinations. These two methods have been recommended as useful in the assessment of pulmonary edema.^40^Nevertheless, in the reporting LUS findings, it will be important that both continuous and categorical data are standardized to present LUS measures ( *e.g* . number of lung regions) to facilitate comparison of results across HF studies. The included studies in the present work indicated the prognostic value of B-lines in both in- and outpatients with HF. However, as they had different outcomes of interest (hospitalization due to HF *versus* composite outcomes of hospitalization and mortality) and different clinical follow-up periods (3 *versus* 6 months), there is a slight difference in the reported optimal cut-off point for B-lines, however, they ranged between 15 and 30. Large randomized controlled trials are required to investigate to what extent the use of LUS would benefit HF patients. Moreover, more studies are needed to find out whether LUS could be applied to identify different phenotypes of patients with HF and to be tailored to the individual patient’s needs.

### Limitations

By design, our analysis did not allow the demonstration of the superiority of B-lines compared to other established biomarkers of HF, such as the NYHA class, NT-proBNP, or 6-min walk test, nor did we evaluate the incremental prognostic value of B-lines over established markers for congestion. Moreover, to our best knowledge, although we are providing the first review and meta-analysis of B-lines in patients with HF, further studies are needed for the optimal treatment of patients with HF with regard to the integrative value of B-lines associated with BNP or risk factors. Thirdly, substantial heterogeneity in this review and meta-analysis among studies indeed existed. The included articles with different patients’ characteristics, B-lines quantification, and risk of bias may contribute to heterogeneity across studies. Also, the number of patients included in our meta-analysis was relatively small, which may have an impact on the exact quantification of the prognostic value of B-lines. In addition, the included studies considered different outcomes. Only one study^24^provided B-lines values both at admission and discharge for combined outcomes of all-cause mortality or HF hospitalization. It would be interesting to examine the changes between the numbers or positions of B-lines at admission and before discharge.

## Conclusions

The present meta-analysis demonstrated that the B-lines could predict all-cause mortality and HF hospitalizations in patients with HF. Further large randomized controlled trials are needed to explore whether dealing with B-lines would improve the prognosis in clinical settings.
